# Discontinuation of the PACE Plus trial: problems in patient recruitment in general practice

**DOI:** 10.1186/s12891-018-2063-1

**Published:** 2018-05-14

**Authors:** M. Schreijenberg, P. A. J. Luijsterburg, Y. D. M. Van Trier, D. Rizopoulos, M. A. Koopmanschap, L. Voogt, C. G. Maher, B. W. Koes

**Affiliations:** 1 Department of General Practice, Erasmus MC, University Medical Center, PO box 2040, 3000 CA Rotterdam, The Netherlands; 2 Department of Biostatistics, Erasmus MC, University Medical Center, PO box 2040, 3000 CA Rotterdam, The Netherlands; 3 Department of Health Policy and Management/iMTA, Erasmus University Rotterdam, PO Box 1738, 3000 DR Rotterdam, The Netherlands; 4 Dutch Association for Back Pain Patients ‘The Spine’, Bentinckstraat 21, Lichtenvoorde, The Netherlands; 5 Sydney School of Public Health, Sydney Medical School, The University of Sydney, PO Box M179, Sydney, NSW 2050 Australia

## Abstract

**Background:**

The PACE Plus trial was a multi-center, double-blinded, superiority randomized controlled trial (RCT) conducted in patients from Dutch general practice to investigate the efficacy of paracetamol and NSAIDs in acute non-specific low back pain (LBP). Because insufficient numbers of patients could be recruited (only four out of the required 800 patients could be recruited over a period of 6 months), the trial was prematurely terminated in February 2017, 6 months after the start of recruitment. This article aims to transparently communicate the discontinuation of PACE Plus and to make recommendations for future studies.

**Methods:**

General Practitioners (GPs) from 36 participating practices received a one-question survey in which they were asked to give the three most important factors that in their opinion contributed to failure of patient recruitment.

**Results:**

GPs of 33 out of 36 (92%) participating practices sent a response. A total of 81 factors were reported. These have been categorized into patient factors (26 out of 81 comments, 32%), GP factors (39 out of 81 comments, 48%) and research factors (16 out of 81 comments, 20%).

**Discussion:**

Patient recruitment in the PACE Plus trial may have failed due to inefficient medication distribution, recruitment of incident rather than prevalent cases, a design that was too complicated, adequate self-management of LBP, patient expectations different from the trial’s scope and lack of time of participating GPs. Substantial differences in design may explain why the preceding PACE trial did manage to successfully complete patient recruitment.

**Conclusion:**

Although the PACE Plus trial was terminated as a result of insufficient patient inclusion, the research questions addressed in this trial remain relevant but unanswered. We hope that lessons learned from the discontinuation of PACE Plus and corresponding recommendations may be helpful in the design of upcoming research projects in LBP in general practice.

**Trial registration:**

Dutch Trial Registration NTR6089, registered September 14th 2016.

## Background

The PACE Plus trial was conducted in Dutch general practice to investigate the efficacy of paracetamol and NSAIDs in acute non-specific low back pain (LBP) [1]. The study design was a multi-center, placebo-blinded, superiority randomized controlled trial (RCT). The two main aims of this RCT were to replicate the comparison between paracetamol and placebo as done in the PACE trial [2–4] and to compare the efficacy of paracetamol with diclofenac and advice only. The study protocol was published [1] and was prospectively registered (Dutch Trial Registration NTR6089, registered September 14th 2016). The Erasmus MC Medical Research and Ethics Committee (MREC) has granted approval for the PACE Plus trial (NL54941.078.16). In short, our intention was to recruit 800 patients with acute LBP from Dutch general practices, who would be randomized across four treatment groups (paracetamol, diclofenac, placebo or advice only) and would be followed for 12 weeks. Inclusion and exclusion criteria for patient recruitment in the PACE Plus trial can be found in Table 1.

**Table 1:** Inclusion and Exclusion criteria for patient recruitment in the PACE Plus trial

Inclusion criteria	Exclusion criteria
• Age between 18 and 60 years; • Low back pain of less than 6 weeks duration; • Primary complaint of pain between the 12th rib and buttock crease; • Experiencing a new episode of low back pain, preceded by a period of at least 1 month without low back pain; • Low back pain severe enough to cause at least moderate pain (≥4 on 0–10 numerical rating scale (NRS)).	• Known or suspected serious spinal pathology; • Currently taking recommended regular doses of analgesics, including paracetamol or diclofenac; • Spinal surgery within the preceding 6 months; • Serious comorbidities preventing prescription of paracetamol or diclofenac; • Use of medication interacting with paracetamol or diclofenac; • Known intolerance for paracetamol or diclofenac; • Pregnancy or planning to become pregnant during the treatment period.

From May 2016, General Practitioners (GPs) were recruited for participation in the trial. Initially, GPs who had experience with patient recruitment in studies conducted by the Erasmus Medical Center (EMC) Department of General Practice were approached for participation. As a second step in the recruitment of GPs, local GPs from the provinces of Zuid-Holland, Noord-Brabant and Zeeland and GPs who were specializing in musculoskeletal disorders at the EMC were approached for participation. Finally, GP residents in their last year of training were asked to participate as part of their training program.

Recruitment of eligible patients for the PACE Plus trial started in September 2016. During the first 10 weeks of the inclusion period of the trial, a total of 79 GPs from 26 practices participated in the trial; GPs from 11 of these practices (42%) had participated in other studies of the EMC Department of General Practice. Twenty-two patients were referred for participation in the trial by a total number of 12 practices. Four out of these 22 patients (18%) could be included in the trial. Eighteen out of 22 (82%) referred patients were excluded; nine patients did not meet inclusion criteria, nine patients declined to participate in the trial after being informed by a research assistant over the phone. Reasons why referred patients did not meet inclusion criteria are presented in Table 2. Shortly after inclusion, the first included patient declined further participation and was lost to follow-up.

**Table 2:** Patient referral and inclusion and exclusion in the PACE Plus trial

Trial period	Before design modification	After design modification	Total
Number of participating GPs (number of participating practices)	**79 (26)**	**96 (36)**	**96 (36)**
Referrals (number of referring practices)	**22 (12)**	**9 (6)**	**31 (15)**
Exclusions (% of referrals)	**18 (82%)**	**9 (100%)**	**27 (87%)**
Patient did not meet inclusion criteria	9	4	13
Intake of study medicines before inclusion	5	1	6
Pain score (NRS 0–10) < 3	1	1	2
Specific cause of low back pain	0	1	1
Age > 60 years	1	0	1
Comorbidity/co-medication with interaction	1	1	2
Insufficient knowledge of Dutch language	1	0	1
Patient declined to participate	9	5	14
Inclusions (% of referrals)	**4 (18%)**	**0 (0%)**	**4 (13%)**

In November 2016, the trial was temporarily suspended due to insufficient patient recruitment and the ‘Advice only’-group was removed from the design after approval from the Medical Research and Ethics Committee (MREC) of the Erasmus MC and the funding party (ZonMw), because a majority of participating GPs reported that their patients with LBP did not accept the 25% chance of receiving no medication whatsoever. The ‘Advice only’-group was perceived by many patients as well as participating GPs as doing nothing. As a result of the design modification, two of the four included patients were censored from the trial because they had been randomized to the ‘Advice-only group’.

After the design modification, a total of 96 GPs from 36 practices participated in the trial; GPs from 13 of these practices (36%) had participated in other studies of the EMC Department of General Practice. Nine more patients were referred for participation in the trial by a total number of six practices. None of the nine patients could be included in the trial; four patients did not meet inclusion criteria and five patients declined participation after being informed over the phone by a research assistant.

Because insufficient numbers of patients could be included despite the study design modification, the PACE Plus trial was terminated in February 2017, approximately 6 months after the start of recruitment. To investigate the underlying reasons for termination of this RCT, GPs from all participating practices were sent a survey. This publication has two aims: firstly, to provide transparent communication about our unsuccessful patient recruitment, including results from the GP survey and secondly, to make recommendations for future researchers in this field of study in order to avoid the problems encountered in this trial.

## Methods

After the PACE Plus trial was discontinued, a letter explaining the trial had been terminated because of insufficient patient recruitment was sent to all local research collaborators (one GP for each participating practice, *n* = 36). Attached to this letter was a form with 3 blank answer boxes and a single question: “In your opinion, what are the (3) most important reasons why patient recruitment failed?”. GPs were requested to return their answer to this question in a pre-paid envelope that was provided. Reminders were sent 2 and 3 months after the original letter to GPs who had not yet responded to the survey.

MS extracted all responses into Microsoft Excel 2010 as individual reasons. BK, PL and MS created 3 reason categories: patient factors (i.e. factors related to patient expectations and coping mechanisms), GP factors (i.e. factors related to presentation of patients in clinical practice and organization of care) and Research factors (i.e. factors related to trial design and organization). PL and MS categorized all reasons into one of these categories. MS computed percentages using Microsoft Excel 2010 and interpreted initial results. BK and PL checked these computations and interpretation. For categories with a minimum of 8 responses (10% of total reasons), BK and MS selected quotes that represented the opinions of multiple GPs for that specific category. Quotes were translated from Dutch to English by BK and MS.

## Results

Twenty-six out of 36 GPs responded to the survey after the first letter (19 returned the original filled-out form by post, seven sent an e-mail with their opinion). Six out of the remaining 10 GPs responded after the first reminder (four by post, two by e-mail). After a final reminder, one of the remaining four GPs responded to the question by e-mail. In total, 33 out of 36 practices (94%) sent a response. Not all respondents sent back exactly three factors, this ranged from one to four factors per response. Responses were usually formulated as short sentences. A total of 81 factors were reported (Table 3). These have been categorized into patient factors (26 out of 81 comments, 32%), GP factors (39 out of 81 comments, 48%) and research factors (16 out of 81 comments, 20%).

**Table 3:** Results of survey amongst 33 participating GPs

GP opinions on why recruitment failed in the PACE Plus trial	Total number of comments (% of total comments)
1. Patient factors:	26 (32%)
1.1 Patient had other expectations when seeking care for low back pain	14
1.2 Patients were confident they could self-manage their low back pain	7
1.3 Patient declined participation	5
2. GP factors:	39 (48%)
2.1 Insufficient number of patients meeting criteria were seen in practice or spoken to on the telephone	20
2.2 Lack of time or trial forgotten because of other tasks	14
2.3 The trial had just started or had not yet started in the practice	3
2.4 Not all employees of the practice were sufficiently informed about the trial	2
3. Research factors:	16 (20%)
3.1 Medication distribution procedure too complicated	7
3.2 Research question and design irrelevant for clinical practice	4
3.3 Inclusion and exclusion criteria too restrictive	2
3.4 Research logistics disturb usual clinical care	2
3.5 Problems in communication with research department	1
Total number of comments from 33 GPs:	81 (100%)

Most of the comments about patient factors stated that patients had other expectations when seeking care for LBP than participating in a trial (14 out of 26 comments). Examples of these expectations from the survey were patients asking either for alternatives for paracetamol or for stronger pain medication than NSAIDs and patients requesting further diagnostics by x-ray. One GP wrote: “*The study went against expectations of patients and doctors. The idea not to take or prescribe pain medication when someone is in pain requires abstract reasoning. GPs aren’t happy to dismiss paracetamol, too much time was invested to promote the usefulness of this drug. This means there is a lose-lose situation; both the patient and the GP lose in this trial (at least from a superficial point of view)*”. Other patient factors mentioned by GPs were that patients felt confident they could self-manage their LBPs (using validated online patient information such as the patient information website of the Dutch College of General Practitioners (NHG) [5] or using direct access to physiotherapy, seven out of 26 comments) and patients declining participation in the trial directly in the practice (five out of 26 comments).

Nearly a quarter of all comments mentioned insufficient numbers of patients meeting the inclusion criteria for the PACE Plus trial presenting in the practice or on the phone (20 comments). The quote best capturing this stated: “*I personally see few people in my practice that meet the inclusion criteria. Age, duration of complaints, etcetera. The GP’s assistant solves a lot of cases; those patients could otherwise have participated in the trial*”. Another reason that was often stated was lack of time due to patient care and administration tasks (14 comments). Several GPs mentioned forgetting about the trial because of the high workload; one respondent wrote: *“It’s very hectic! I only remembered to ask my patient to participate after he’d already left”.* Other GP factors considered organizational issues in the GP practices: two GPs stated that not all employees in the practice were sufficiently informed about the trial, three GPs had only just or not yet started participating in the trial.

Research factors reported could be related to both study design choices and trial organization by the research department. Apart from the GPs mentioning insufficient numbers of patients presenting in their practice, two GPs explicitly stated that inclusion and exclusion criteria were too restrictive to be realistic. Seven GPs found the medication distribution procedure too complicated and the subsequent time delay before the patient received medication unacceptable. Four GPs believed the trial research question was irrelevant for clinical practice. Two GPs stated that the trial design caused disturbance of usual clinical care. Finally, one GP mentioned that communication with the research department was not clear enough.

## Discussion

Termination of the PACE Plus trial may be attributable to research logistics and design, patient related and GP related factors. We will discuss these factors considering the survey described above as well as reflecting on design choices made in this trial’s predecessor, the PACE trial, which did manage to successfully recruit 1650 patients with comparable complaints.

In retrospect, an important weakness in the logistics of PACE Plus was the complicated medication distribution procedure, as mentioned by several GPs in the survey. Patients could only be randomized once informed consent was signed and the baseline questionnaire had been filled out; very often, patients could not find the time to do this immediately after referral, which meant randomization and preparation of a medication pack would be delayed. As GP practices participating in the trial were spread across three Dutch provinces, 24-h postal delivery was used to get medication packs to participants. In practice, this meant that patients who were in pain had to wait at least 24 to 48 h before receiving medication; in contrast, if patients declined to participate in the trial and asked their GP for a prescription, they would usually be able to pick up pain medication at their local pharmacy within an hour. Alternatively, patients could buy paracetamol and NSAIDs as over-the-counter medication without a prescription. Although our medication distribution procedure did fit within the limitations of Dutch law on medical research in humans, we have underestimated the potential for delay to arise in practice, the discomfort this meant for the participants and the unfavorable position of the trial in comparison to conventional treatment options.

During the design phase of PACE Plus, an alternative medication distribution procedure was considered. In this scenario, GPs would be asked to inform patients about the trial, collect informed consent, randomize patients and give them a medication pack immediately. However, under Dutch law on medical research in humans, this meant medication packs would have to be stored in a locked, temperature controlled environment, for which extra records would have to be kept by participating GPs. Although this scenario more closely resembled clinical practice and diminished delay, the procedure was dismissed because it would ask a substantially larger investment of time of participating GPs (who were already on a very tight schedule) and would require purchasing special medication storage equipment for all participating practices, for which trial budget did not allow.

Apart from logistics, alternative target populations were also considered during design of the trial. We chose to recruit patients with a new episode of acute LBP (incident cases) as opposed to prevalent cases of acute LBP (less than 6 weeks of pain) for two reasons. Firstly, our main aim was to replicate the PACE trial, which used incident cases. Secondly, many patients with prevalent LBP would already be using recommended doses of paracetamol or NSAIDs and would therefore be ineligible for participation in the trial. A more feasible alternative might have been to recruit patients with chronic LBP, but this of course would mean investigating a completely different research question and a design with a much longer follow-up period. Therefore, although more challenging than the alternatives, recruitment of incident cases of acute LBP seemed the most appropriate choice considering the aim of our trial. The discontinuation of this trial supports the previous finding that recruiting incident cases during the GP’s consultation is associated with a lower probability of complete and timely patient recruitment [6]. For future research, other designs such as an RCT embedded in a cohort of patients with recurrent LBP could be considered as an alternative to recruitment of incident cases during the first consultation.

Another design choice that may have impacted the feasibility of this trial was the objective to both repeat PACE and explore the alternatives to paracetamol in one trial. Not only did this mean that double the number of patients had to be recruited than when comparing just paracetamol and placebo, it also meant that the trial was more complicated to explain to both participating GPs and eligible patients. In hindsight, it might have been better to focus on one of our objectives and design the trial accordingly.

In terms of patient factors, effective self-management of LBP may have had an impact on patient recruitment. This is supported by the fact that the most important reason for exclusion during PACE Plus was patients already taking one of the study medicines; furthermore, several GPs mentioned in the survey their patients were confident they could self-manage their LBP. Several societal developments could have contributed to this improved self-management. Firstly, since 2006, patients have access to physiotherapy without referral from a GP, although research suggests this does not influence the number of GP visits [7]. Secondly, it was demonstrated that the introduction of a patient information website of the NHG [5] has led to a decrease in healthcare usage of 12% [8]. Finally, both paracetamol and NSAIDs are available without a doctor’s prescription, as they are registered as over-the-counter medications in the Netherlands.

In PACE Plus, 14 of 27 excluded patients declined to participate in the trial. This is highly related to the comments of GPs that patients had other expectations and that patients declined participation. The trial did not provide any new treatment or in fact, any intervention that the patient could not obtain over-the-counter as mentioned above; instead, it relied on altruism of patients to answer the research question. The reason why patients declined to participate may have been because the underlying problem and research question were not considered relevant enough by many patients, as was mentioned in the survey by four GPs. This may have been avoided by discussing research ideas with a group of acute low back pain patients and taking their specific preferences and expectations for both treatments and outcomes into consideration.

Lack of GP’s time was often mentioned as a factor affecting patient recruitment. This statement appears to reflect recent trends in increasing workload for Dutch GPs because of changes in the national health care system [9]. As a result of this increasing workload, less time is available for participating in clinical research, which affects the feasibility of conducting clinical trials in general practice [10].

GPs participating in the PACE Plus trial reported a lower incidence of acute LBP than was initially expected based on incidence figures reported in the NHG practice guideline [11]. This may have been because of Lasagna’s law [6, 12, 13], the phenomenon that researchers overestimate the number of available patients meeting inclusion and exclusion criteria of a trial (originally formulated as “the incidence of patient availability sharply decreases when a clinical trial begins”).

Considering PACE Plus investigated a highly similar patient group and similar interventions to the original PACE trial, we looked into differences between the two studies that may explain why PACE successfully completed patient recruitment while PACE Plus failed to do so. Firstly, the total budget in the PACE trial was substantially higher than in PACE Plus, which meant that in PACE, three fulltime research assistants could be employed as opposed to 1.2 fulltime equivalent in PACE Plus. Furthermore, both GP’s and participants could be reimbursed for their time invested in the trial in PACE, whereas there was no compensation in PACE Plus. Other strategies used in PACE but omitted in PACE Plus included conducting a pilot trial and rewarding participating GPs with Mandatory Continuing Education (MCE) points. In PACE, a ‘novel’ treatment (modified release paracetamol) was investigated that could potentially have been added to conventional treatment options, whereas in PACE Plus, two of the treatments were somewhat controversial both for GPs and patients (diclofenac and no medication); additionally, some GPs feared losing paracetamol as a treatment option. Finally, as opposed to the complex medication distribution procedure used in PACE Plus, medication was allowed to be directly provided by the GP preventing delay in patients commencing their pain relief medicine.

## Conclusion

Although the PACE Plus trial was terminated as a result of insufficient patient inclusion, the research questions addressed in this trial remain relevant but unanswered. This is especially true in light of recent international LBP guidelines [14–17], in which the use of any medication for LBP is discouraged. Lessons learned from the discontinuation of PACE Plus and corresponding recommendations have been summarized in Table 4. We hope that these lessons and recommendations may be helpful in the design of upcoming research projects in LBP in general practice.

**Table 4:** Lessons learned from discontinuation of the PACE Plus trial and corresponding recommendations for future research

Lessons learned	Recommendations for future research
• Even though treatment distribution follows legislation and works on paper, they may have issues in practice that affect patients. • Asking GPs to recruit incident cases during the first consultation seems unlikely to be successful considering the current workload in general practice. • Attempting to answer several research questions at once not only requires more patients but is also more complicated to explain to GPs and potential participants. • Interests and expectations of patients can collide with scientifically interesting questions in practice. • The number of available patients meeting inclusion criteria is easily overestimated (Lasagna’s Law). • Negative perception of trial treatment may influence participation of both GPs and patients.	• Keep treatment distribution as simple as possible and provide an attractive alternative to conventional therapy. • Try to answer your research question in prevalent cases or use alternative designs such as a trial within a cohort study. Take into account reimbursement of GPs in grant application and budgeting (especially if you do end up recruiting incident cases). • Choose your most important research question and design your trial to be as simple as possible. • Before starting a trial, ask a patient panel for their preferences and expectations of treatments and outcomes. Ask GPs if they know of any reservations about treatments you consider using. • Take Lasagna’s law into account when planning your trial. • Consider conducting a pilot trial and taking part in Mandatory Continuous Education.

## Abbreviations

EMC: Erasmus Medical Center
GP: General Practitioner
LBP: Low back pain
MCE: Mandatory Continuing Education
MREC: Medical Research and Ethics Committee
NHG: Dutch College of General Practitioners
NRS: Numerical rating scale
RCT: Randomized controlled trial
ZonMw: The Netherlands Organisation for Health Research and Development
